# Cerebellar ataxia, neuropathy and vestibular areflexia syndrome (CANVAS): from clinical diagnosis towards genetic testing

**DOI:** 10.1515/medgen-2021-2098

**Published:** 2022-01-12

**Authors:** Andreas Thieme, Christel Depienne, Dagmar Timmann

**Affiliations:** Department of Neurology and Center for Translational Neuro- and Behavioral Sciences (C-TNBS), Essen University Hospital, Hufelandstraße 55, 45147 Essen, Germany; Institute for Human Genetics, Essen University Hospital, Hufelandstraße 55, 45147 Essen, Germany

**Keywords:** late-onset recessive ataxia, sensory ataxia, RFC1 repeat expansion

## Abstract

The cerebellar ataxia, neuropathy and vestibular areflexia syndrome (CANVAS) is a late-onset and recessively inherited ataxia. For many years, CANVAS has been diagnosed based on the clinical phenotype. Only recently, a large biallelic pentanucleotide repeat expansion in the replication factor C subunit 1 (*RFC1*) gene has been identified as the underlying genetic cause for the large majority of CANVAS cases. Subsequently, other phenotypes such as ataxia with chronic cough, incomplete CANVAS and MSA-C-like phenotypes have been associated with biallelic *RFC1* repeat expansions. Because of this heterogeneity it has been suggested to change the name of the disease to “RFC1 disease”. Chronic cough is characteristic and can precede neurological symptoms by years or decades. In the neurological examination signs of cerebellar, sensory, and vestibular ataxia are frequently observed. Nerve conduction studies usually show absent or markedly reduced sensory nerve action potentials. On brain MRI cerebellar degeneration and spinal cord alterations are common. In later disease stages more widespread neurodegeneration with additional involvement of the brainstem and basal ganglia is possible. As yet, the exact incidence of *RFC1*-associated neurological diseases remains uncertain although first studies suggest that *RFC1*-related ataxia is common. Moreover, the pathophysiological mechanisms caused by the large biallelic pentanucleotide repeat expansions in *RFC1* remain elusive. Future molecular and genetic research as well as natural history studies are highly desirable to pave the way towards personalized treatment approaches.

## Introduction

Cerebellar ataxia, neuropathy and vestibular areflexia syndrome (CANVAS) is a late-onset ataxia [1]. In the 1990s, the coexistence of ataxia and vestibulopathy was first reported [2], [3], [4], [5] and confirmed in subsequent case series. Additionally, neuropathy has been reported in some patients [6], [7], [8], [9]. In 2011, *CANVAS* was introduced as a new entity [1]. Both sporadic and familial cases have been observed with siblings being affected while parents were healthy. Hence, a recessive mode of inheritance has long been suspected [10], [11], [12]. In 2019, a biallelic intronic repeat expansion in the replication factor C subunit 1 (*RFC1*) gene was identified as the underlying mutation responsible for a large portion of CANVAS cases [13], [14], particularly in populations of European descent [15], [16]. Based on these findings, *RFC1* expansion disorder might be a common and possibly underdiagnosed cause of adult-onset ataxia in some populations. This review summarizes the current knowledge on CANVAS respectively *RFC1* repeat expansion disorder with a focus on clinical presentation, findings in diagnostic tests, molecular genetics and pathophysiological mechanisms.

## Clinical presentation

CANVAS is a late-onset, slowly progressive disorder. The average annual disease progression rate measured by the *Scale for the Assessment and Rating of Ataxia* (*SARA*) lies within the range of other hereditary ataxias [17], [18], [19]. The acronym “*CANVAS*” stands for the cardinal features: cerebellar ataxia, sensory neuropathy and bilateral vestibular areflexia syndrome [1], [10]. Histopathological examinations following the initial description of CANVAS have shown that one key feature is rather a neuronopathy (ganglionopathy) than a neuropathy [20], [21]. Onset usually occurs between the age of 50 and 60 years (range: 19–82) [1], [13], [22], [23]. The most frequent initial symptom is progressive balance loss and unstable gait, especially in the dark when compensatory visual control is missing (∼100 % of all patients). The next common symptoms are sensory deficits (∼75 %) including hyp- and dysesthesia, allodynia and neuropathic pain, postural instability and dysarthria (∼50 % each) [1], [10], [11], [13], [16], [22], [24]. Chronic, non-productive cough is typical (∼65 %) and often precedes the onset of imbalance and somatosensory impairment by years, in some cases by decades (median age of onset: 35 years) [16], [22], [23]. Autonomic dysfunction is frequent (∼60 %) as well. In most patients, it is mild and includes orthostatic hypotension, chronic constipation or fecal incontinence, urinary and/or erectile dysfunction, gastroesophageal reflux and hypohidrosis. Furthermore, dysphagia, oscillopsia, bradykinesia (∼30 % each) and cognitive impairment (∼25 %) are quite common while REM-sleep behavioral disorders, sleep apnea, progressive supranuclear palsy, slowing of saccades, pyramidal tract involvement and hyperkinetic movement disorders are not typical and only rarely occur [13], [16], [22], [23], [24], [25].

## Molecular genetics

**Table 1:** *RFC1* allele confirmations and their association to CANVAS.

**Allele confirmations** ***not associated*** **with CANVAS and their frequency**			
**Frequency (percentages indicate allelic distribution)**	**Allele**		**Study**
75.5 %	(AAAAG)_11_		[13]
13.0 %	(AAAAG)_exp_		[13]
7.9 %	(AAAGG)_exp_		[13]
2.1 %	(AAGAG)_exp_		[26]
**Allele confirmations** ***associated*** **with CANVAS and their frequency**			
**Frequency (percentages indicate allelic distribution)**	**Allele 1**	**Allele 2**	**Study**
0.7–6.8 %	(AAGGG)_exp_	(AAGGG)_exp_	[13], [14], [15]
1 patient	(AAGGG)_exp_	(AAAGG)_exp_	[14]
1 patient	(ACAGG)_exp_	(ACAGG)_exp_	[25]
2 patients	(AGAGG)_exp_	(AGAGG)_exp_	[26]
3 patients	(ACAGG)_exp_	(ACAGG)_exp_	[27]
21 patients versus 7 healthy carriers	(AAGAG)_exp_	(AAGAG)_exp_	[26]

Despite the description of multiple families arguing for a recessive inheritance [10], [11], [22], [23], [24], the genetic cause of CANVAS had remained unknown for several years. In 2019 – eight years after the initial description of CANVAS – a biallelic pentanucleotide repeat expansion in the *RFC1* gene could finally be identified in a large number of familial and sporadic CANVAS cases. This discovery was consecutive to the identification of a unique locus on chromosome 4p14 using genome-wide linkage analysis. In the linked region, two different groups identified an intronic repeat expansion composed of AAGGG motifs in the *RFC1* gene using a combination of whole genome sequencing, repeat-primed analysis, long-read sequencing and/or Southern blot [13], [14]. These pathogenic repeat expansions differ both in size and nucleotide sequence from the (AAAAG)_11_ reference allele. Expansion size ranges from ∼400 to ∼2,000 repeats (i. e., 2 to 10 kb) on average [13], [14], and most patients with AAGGG expansion shared the same haplotype which is thought to have originated in Europe about 25,000 years ago – most likely due to a founder effect. But contrary to other repeat expansion disorders, no particular association between the expansion length and the age at onset has been observed so far.

Interestingly, the region of intron 2 of *RFC1* where the AAGGG repeat expansion occurs is rich in repeated elements including Alu repeats. Furthermore, the study of this repeat region (chr4: 39350045–39350103) in unaffected and affected individuals has revealed a huge variability in repeat motif and size, with at least four additional motifs (AAAGG, AAGAG, AGAGG and ACAGG) existing in addition to the non-pathogenic AAAAG and pathogenic AAGGG repeats (see Table 1). Non-pathogenic AAAAG repeat expansions are present in 13 % of unaffected individuals and monoallelic pathogenic AAGGG repeats could be present in up to 1–6 % of the population [13], [14], [15], [26]. Most of the alternative motifs and conformations are not yet reliably classified as pathogenic or benign and their consequence in human pathology is still largely unknown. Moreover, the existing tests used to assess the presence of the expansion do not usually provide information on its length and structure, which might be required elements to correctly classify some alleles. So far, only biallelic *RFC1* repeat expansions are considered pathogenic.

The estimated prevalence at birth of biallelic carriers lies between 1:20,000 and 1:400 (based on calculations for the most common pathological (AAGGG)_exp_ allele) [13]. For comparison, the incidence of Friedreich’s ataxia (FRDA), which is also recessively inherited, is much lower, between 2 and 4:100,000. These data along with the high prevalence of biallelic *RFC1* expansion carriers amongst ataxic patients (up to 92 % in patients with a full CANVAS phenotype) [13] suggest that CANVAS may be a comparatively frequent and underdiagnosed form of ataxia – especially in populations of European descent. Lower incidences have been reported in other studies including patients of European ancestry (e. g., 0.6–4 % in [26]) or other populations including Japanese populations (8.5–12 % in [25]), while so far no *RFC1* expansion carriers have been identified in a Chinese cohort [17]. These data further support the hypothesis of a founder effect with origin in Europe, but also show that *RFC1* expansions exist in populations with other genetic backgrounds. Besides, large studies described a few patients who did not share the common CANVAS haplotype, arguing for the existence of independent expansion events [13], [14].

In one of the largest studies performed so far, *RFC1* expansions were identified in ∼92 % of patients with a full CANVAS phenotype, ∼63 % of patients with ataxia and neuronopathy and ∼22 % of patients with ataxia only [13]. These findings suggest that other causative mutations are likely to exist in at least ∼8 % of CANVAS patients [14], [15], [16]. To our knowledge, only one dominantly inherited missense variant in the *ELF2* gene has yet been reported in a British family with a phenotype compatible with CANVAS. *In vitro* experiments showed that the *ELF2* mutation led to an increased expression of Ataxin-2 (SCA2) as well as a reduced expression of ELOVL5 (SCA38), a result establishing links between ELF2-disorder and other ataxia subtypes [28].

## Pathophysiology

As yet, the pathophysiological mechanisms by which biallelic expansions lead to CANVAS remain largely unknown. The *RFC1* gene encodes the replication factor C subunit 1, a protein involved in DNA repair and replication. Interestingly, a few other hereditary disorders associated with ataxia and neuropathy such as ataxia with oculomotor apraxia type 1 (AOA1) or ataxia telangiectasia (AT) arise from mutations in genes that encode proteins involved in DNA repair and replication [29]. Surprisingly however, fibroblasts from biallelic *RFC1* expansion carriers do not show an increased susceptibility to DNA damage *in vitro* [13]. Recessive disorders are usually due to a loss-of-function of the gene where the mutation occurs. This also applies to expansion disorders including FRDA [29], [30]. However, in the case of biallelic *RFC1* expansions, the mRNA and protein expression levels of RFC1 are normal, and expansions do not alter splicing of exons 2 and 3. These findings speak against a loss-of-function of the *RFC1* gene. Moreover, no clear effect has been seen on the expression of neighboring or more distant genes. RNA molecules filled with repeats have not been detected but a slight increase of RNA molecules retaining intron 2 has been observed in patients’ cells. Based on this finding, RNA toxicity and/or repeat-associated non-AUG (RAN) translation generating toxic repeat-encoded polypeptides have been discussed as possible pathophysiological mechanisms [13]. However, both mechanisms are usually associated with dominant expansion disorders, as illustrated by other forms of spinocerebellar ataxias related to intronic repeat expansions such as spinocerebellar ataxias (SCA) 8, 10, 12, 31, 36 and 37 [31], [32] and familial adult myoclonic epilepsy (FAME), another dominant repeat expansion disorder linked to TTTCA repeat expansions [33].

However, there are several lines of evidence supporting the repeat expansion as pathogenic. First, the discovery of different expanded repeat motifs specifically associated with CANVAS when present on both alleles (see Table 1) supports this hypothesis [26]. Second, the average size of the “healthy alleles” ranges from 15 to 200 for (AAAAG)_exp_ and from 40 to 1,000 repeats for (AAAGG)_exp_, whereas the size of (AAGGG)_exp_ pathological alleles is considerably larger, ranging from 400 to 2,000 repeats [13]. The missing correlation between repeat length and disease onset or severity supports the hypothesis that repeat sequence rather than length may drive the pathogenetic alterations [13], [22]. Finally, SCA37 and FAME are also disorders characterized by large pentanucleotide repeat expansions containing a pathogenic motif different than the one normally present at the locus [13], [31], [33].

Despite the still unclear molecular mechanisms, it is well known that neuronal loss takes place in CANVAS. Postmortem histopathological examinations have revealed dorsal root and cranial nerve neuronopathy as well as marked cerebellar Purkinje cell loss. Neuronopathy preferentially affects the cranial nerves V, VII and VIII. In the vestibulocochlear nerve (VIII), atrophy of the vestibular portion is observed while the cochlear portion is preserved. Accordingly, hearing loss is usually not seen clinically in CANVAS patients. Glossopharyngeal and vagal neuronopathy might explain dysphagia and chronic cough. Pharyngeal and upper airway denervation with resulting hypersensitivity and/or disturbed coordination during deglutition are assumed by some authors [11] while others argue that dysfunctional peripheral and brainstem or cerebellar networks involved in regulation of the cough reflex might explain chronic cough [16]. Likewise, dorsal root ganglia and dorsal roots are atrophied. In the dorsal columns of the spinal cord marked loss of myelinated axons is observed which is assumed to result secondary to dorsal root atrophy. Cerebellar Purkinje cell loss particularly affects the vermis and hemispheric crus I and is accompanied by Bergmann layer gliosis. The cerebellar dentate nucleus is spared. Using p62 immunostaining, no pathological cytoplasmic or intranuclear inclusions in the cerebellar cortex have been detected [21], [34]. In skin biopsies of two CANVAS patients, sweat gland denervation has been found, indicating that postganglionic autonomic nerves are affected as well [35].

## Findings in diagnostic tests and differential diagnoses

Each of the three CANVAS defining features may cause ataxia alone, that is, cerebellar, vestibular and sensory ataxia [24]. CANVAS may be incomplete and if a full CANVAS occurs, there is no distinct sequence to the onset of the cardinal features. The final component of the diagnostic triad may develop after more than 10 years. However, early involvement of sensory neurons is a common finding [21], [22], [24].

Note that several symptoms of CANVAS overlap with those of other neurodegenerative disorders and that atypical presentations linked to *RFC1* expansion are common. Therefore, some authors prefer a genotypic definition as “*RFC1 expansion disorder*” rather than the phenotypic description of “CANVAS” [16]. Variants with only ataxia and chronic cough or phenotypes similar to those of multiple system atrophy – cerebellar type (MSA-C) – exist. Long disease duration, milder urogenital involvement and sensory neuronopathy may help to distinguish CANVAS from MSA-C [23], [36]. Polyneuropathy is not a frequent feature of MSA-C; it appears to be more frequent in MSA-P and it is predominantly a motor axonopathy [37]. Furthermore, pyramidal and extrapyramidal involvement are rare in CANVAS, but occur frequently in MSA (and other neurodegenerative disorders) [1], [10], [16], [22], [24], [38]. Also spinocerebellar ataxias – especially SCA3 – as well as FRDA may have a CANVAS-like phenotype [14]. Aside from ataxia, SCA3 may present with vestibulopathy [39], [40], [41], sensory neuropathy [42] and a pattern of autonomic failure similar to that in CANVAS [23], [36]. Late- or very-late-onset FRDA has many overlapping features with CANVAS as well. Sensory and cerebellar ataxia, sensory neuronopathy and neuropathy as well as affection of the vestibulocochlear nerve are common findings in FRDA. However, hearing is typically intact in CANVAS, whereas hypacusis is common in FRDA [11], [30].

For many years, the diagnosis of CANVAS was made upon the clinical presentation alone [10], [24]. Only since recently, genetic testing for the newly found biallelic *RFC1* repeat expansion is available. It is remarkable that large recent studies found a positive predictive value of up to >90 % for the presence of a biallelic *RFC1* mutation if full CANVAS was present or if afferent ataxia, sensory symptoms and chronic cough coexisted [13], [16]. Nevertheless, the *RFC1* expansion is not detected in all CANVAS patients.

### Neurological examination

In CANVAS, cerebellar dysfunction usually presents as gait and truncal ataxia, oculomotor deficits and dysarthria. While oculomotor deficits often are present at disease onset, dysarthria occurs later [22], [23]. Importantly, gait ataxia is not the expression of cerebellar dysfunction alone. It results from cerebellar, sensory and vestibular dysfunction. Typically, Romberg’s sign is positive due to the neuronopathy and/or secondary dorsal column degeneration [21], [24]. Detailed examination of sensory deficits should be carried out since patchy sensory deficits are more typical for a neuronopathy than symmetrical and distal sensory loss as frequently seen in polyneuropathies [24]. Clinical evidence of vestibular dysfunction comprises a pathological head impulse test (i. e., vestibuloocular reflex [VOR] gain), abnormal dynamic visual acuity and an abnormal visually enhanced VOR (VVOR; doll’s head phenomenon). The latter can easily be detected in a bedside examination by turning a patient’s head slowly (at ∼0.5 Hz) from one side to the other while the patient visually fixates upon a stable target. The test is pathological if the patient’s compensatory eye movements are saccadic rather than smooth [10], [12], [24]. Finally, the absence of perspiration (e. g., patient’s socks are dry) and orthostatic dysregulation (e. g., light-headedness after rising up from the examination bed) may indicate autonomic dysfunction [23], [24], [35].

### Nerve conduction studies

**Figure 1:**
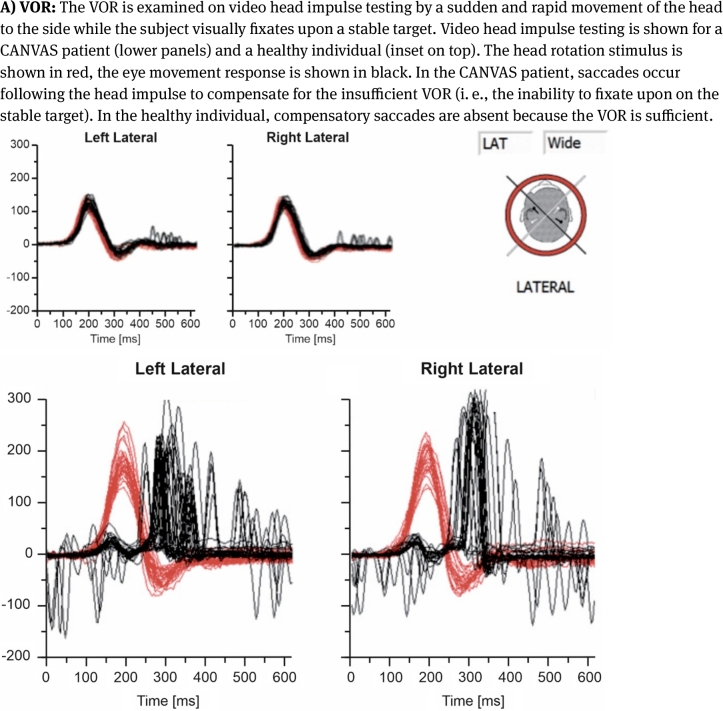

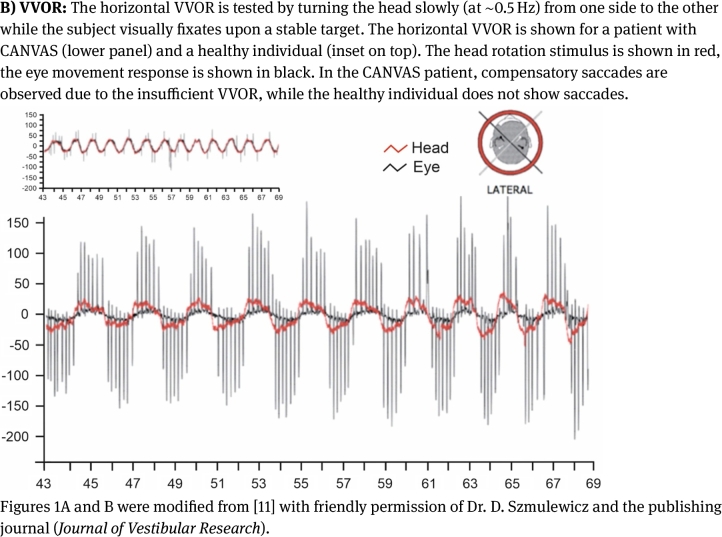
Video assessments of VOR and VVOR in a CANVAS patient.

Nerve conduction studies are highly recommended, particularly, because it has been found that the clinical examination is unreliable for detection of somatosensory deficits in CANVAS. Besides, intact tendon reflexes and pain sensation are not unusual [22], [24]. It has been postulated that sensory neurons innervating muscle spindles (mediating the afferent impulse of tendon reflexes) and Aδ and C (mediating pain) fibers are selectively spared while Aα (mediating proprioception) and Aβ (mediating superficial sensation) sensory neurons are affected [22]. Consistent with this assumption, preserved H reflexes have been found in some studies [43], [44]. In contrast, another study found absent H reflexes in 11 of 14 CANVAS patients [12]. Neuropathic pain which is seen in some patients and sweat gland denervation (= affection of C fibers) contradict the postulated sparing of Aδ and C fibers as well [24], [35]. However, the main electrophysiological finding is uniformly reported in all studies: absent or markedly reduced sensory action potentials in the upper and lower limbs. Often sensory deficits are more severe in the upper limbs. Conduction velocities are either normal or mildly reduced. Mild affection of motor neurons is possible, but the sensory deficit is much more severe [11], [12], [22], [24]. Furthermore, one study reported abnormal somatosensory evoked potentials, not distinguishing between central and peripheral conduction velocities [12]. Blink and masseter reflexes are also abnormal in CANVAS. In contrast, brainstem auditory responses are normal, indicating intact peripheral and central auditory function [12], [21].

### Assessment of autonomic dysfunction

Autonomic dysfunction is frequent in CANVAS. Sudomotor testing (“sweat tests”), tilt table testing, and heart rate and blood pressure recordings during the Valsalva maneuver, deep breathing and standing, as well as the measurement of the cutaneous silent period in the upper and lower limbs have been shown to be useful methods for detection of autonomic deficits in CANVAS [23], [24].

### Investigations of vestibular function

Modalities that can be used to detect abnormal VOR gain include the (video) head impulse test (Fig. 1A), abnormal dynamic visual acuity and abnormal occlusive fundoscopy. Objective evidence of an abnormal VVOR can be detected on high-speed video-oculography, rotational chair testing, and videonystagmography (Fig. 1B), or with magnetic scleral search coil technology. Furthermore, caloric testing is helpful to show vestibular hypofunction [20], [22], [23], [24].

### Brain and spinal cord imaging

**Figure 2:**
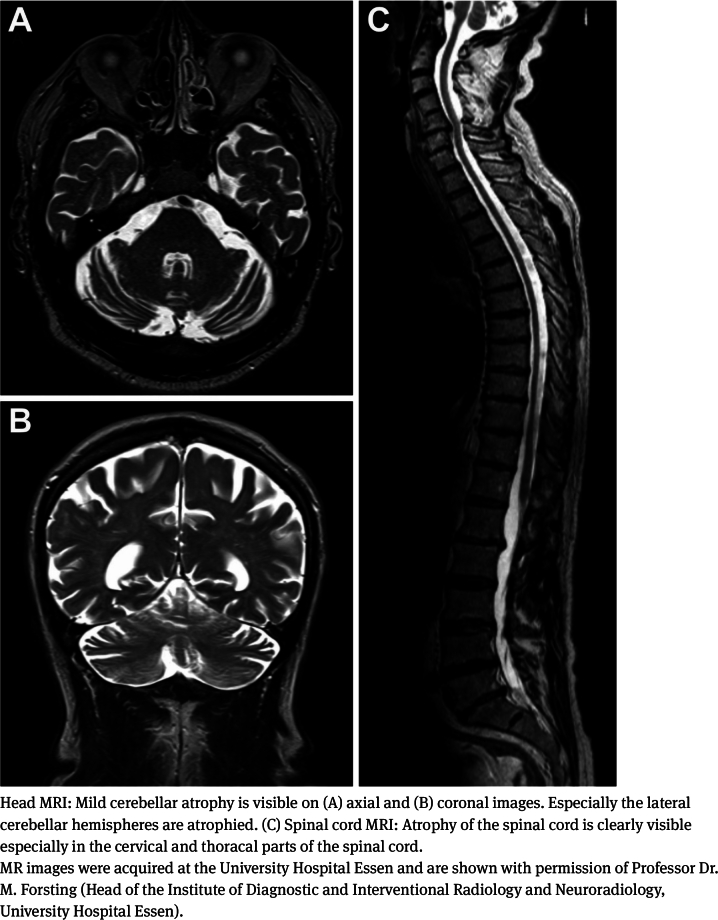
MRI of the head and spinal cord of a genetically confirmed *RFC1* expansion CANVAS patient.

In most CANVAS patients, brain magnetic resonance imaging (MRI) or computed tomography (CT) shows cerebellar atrophy [13], [22]. Early during the disease, cerebellar atrophy may be subtle. Though, cerebellar atrophy may precede cerebellar symptoms. The anterior (vermal lobules I–V) and dorsal vermis (vermal lobules VI, VIIa and VIIb) and the lateral hemispheres (particularly crus I) are primarily affected [10], [11], [22], [23], [24] (see Fig. 2). In one recent study conducted in patients with a more long-standing disease duration, more widespread cerebellar, brainstem and cerebral changes have been reported. Particularly, marked atrophy of the brainstem and the basal ganglia was observed and the diffusivity of the cerebellum, brainstem and cerebral white matter was altered. To a lesser extent also the cerebral cortex showed some atrophy [45]. Moreover, abnormalities on spinal MRI are frequent. One study found a prevalence of 45 % [22]. Atrophy and T2 hyperintensities of the posterior columns are common findings [23], [38], [46].

### Genetic testing

Most patients carry a biallelic intronic *RFC1* pentanucleotide repeat expansion and are of European descent [13], [14], [15], [16], [22]. Fewer cases have been reported in Japan [25], [47], and none have been reported in a Chinese population [17]. Routine testing for *RFC1* repeat expansion is now available and should be performed [13], [14], [15]. In case no *RFC1* repeat expansion can be detected, further genetic testing is recommended, since some hereditary ataxias may present with a CANVAS-like phenotype – particularly, SCA3 and FRDA [24].

## Therapeutic management

To date, a targeted therapy is available only for a few genetic disorders. In fact, the development of such therapies requires a detailed understanding of the underlying pathophysiological mechanisms at a molecular level which yet need to be explored for CANVAS. However, a multidisciplinary symptomatic therapy should be conducted. One of the foremost treatment goals is prevention of falls. There are no data on the incidence of falls in CANVAS patients, but a high risk for falls must be assumed since all senses that contribute to balance may be impaired. Therefore, the fall risk in the patient’s home should be reduced (e. g., placement of grab rails, removal of barriers, etc.) and physiotherapy should be performed [11] – although data only exist for management of cerebellar [48], [49] or vestibular dysfunction alone [50], [51]. Postural hypotension is treated by avoiding drug-induced orthostatic hypotension (caused by diuretics, alpha and beta adrenoreceptor antagonists, etc.), ensuring adequate hydration, wearing elastic stockings and, if necessary, application of fludrocortisone or midodrine. If dysphagia is suspected a speech therapist should be involved and severity should be determined involving video-endoscopic assessments. Most CANVAS patients with dysphagia respond to indirect strategies, i. e., behavioral techniques. Only very few require a modification of food consistency (such as the addition of thickening agents). Finally, sensory symptoms such as allodynia, paresthesia or neuropathic pain may require treatment. Membrane-stabilizing agents such as pregabalin have been found to be effective [11].

## Conclusions and perspective

CANVAS is an only recently described entity. For almost a decade, diagnosis has relied upon clinical findings. Recently, biallelic *RFC1* repeat expansions have been discovered which seem to account for the majority of CANVAS cases and may also account for other phenotypes including ataxia with chronic cough or MSA-C-like disorders. Genetic testing for *RFC1* expansion facilitates the diagnostic work-up. However, several important questions remain unanswered. The exact incidence of CANVAS/*RFC1* expansion disorders in populations with different genetic origins needs to be clarified. Furthermore, the existence of CANVAS cases without *RFC1* expansion raises the possibility of additional genes and/or molecular causes accounting for some CANVAS cases which remain to be discovered. Finally, the pathophysiological mechanisms related to *RFC1* expansions need to be unraveled to inspire the development of targeted therapies in the future.
